# Left Atrial Systolic and Diastolic Dysfunction in Patients with Chronic Constrictive Pericarditis: A Study Using Speckle Tracking and Conventional Echocardiography

**DOI:** 10.1371/journal.pone.0068718

**Published:** 2013-06-18

**Authors:** Shuang Liu, Chunyan Ma, Weidong Ren, Jun Yang, Yan Zhang, Sha Li, Yanbin Cheng

**Affiliations:** 1 Department of Ultrasound, Shengjing Hospital of China Medical University, Shenyang, People’s Republic of China; 2 Department of Cardiovascular Function, First Affiliated Hospital of China Medical University, Shenyang, People’s Republic of China; National Institutes of Health, United States of America

## Abstract

**Background:**

Left atrial (LA) function plays an important role in the maintenance of cardiac output, however, in patients with constrictive pericarditis (CP), whether pericardial restriction and adhesion can lead to LA dysfunction, and the characteristics of LA function remain unclear. The aim of the study is to compare the left atrial (LA) function of patients with CP to that of healthy study participants using speckle tracking echocardiography (STE) and conventional echocardiography.

**Methods and Results:**

Thirty patients with CP and 30 healthy volunteers (controls) were enrolled in the study. The underlying cause of CP was viral pericarditis in 24 (80%) patients and unknown in 6 (20%) patients. The LA maximum volume (Vmax), LA minimal volume (Vmin), and LA volume before atrial contraction (Vpre-a) were measured using biplane modified Simpson’s method. The LA expansion index (LA reservoir function) was determined as follows: ([LAVmax - LAVmin]/LAVmin) ×100. The passive emptying index (LA conduit function) was calculated as follows: ([LAVmax - LAVpre-a]/LAVmax) ×100, and the active emptying index (booster pump function) was calculated as follows: ([LAVpre-a - LAVmin]/LAVpre-a) ×100. All the patients underwent two-dimensional STE. The LA global systolic strain (S), systolic strain rate (SrS), early diastolic strain rate (SrE) and late diastolic strain rate (SrA) were measured. The LA expansion index, passive emptying index, the active emptying index and the LA global S, SrS, SrE, SrA were found to be significantly lower in patients with CP than in the control participants (*P* <0.001). LA function was correlated with the early diastolic velocity of the lateral mitral annulus (*P* <0.05).

**Conclusions:**

Although left ventricular systolic function was preserved in patients with CP, the LA reservoir, conduit, and booster functions were impaired. Pericardial restriction and impairment of the LA myocardium may play an important role in the reduction of LA function in patients with CP.

## Introduction

Chronic constrictive pericarditis (CP) is caused by reduction in the elasticity of the pericardium, which leads to impaired diastolic filling of the heart. The diastolic pressure of the heart, especially the end-diastolic pressure, is elevated because of the constricted pericardium; an enlarged left atrium in patients with chronic constrictive pericarditis is a common finding in clinical practice. Although left atrial (LA) abnormalities are associated with abnormal diastolic function of the left ventricle, the characteristics and pathophysiology of the left atrium in patients with CP is poorly understood.

Measurement of LA volumes, myocardial wall motion, and transmitral flow using conventional two-dimensional (2-D) and Doppler echocardiography can provide important information for the evaluation of LA function. However, these approaches are limited with regard to the evaluation of myocardial performance and have other technical limitations that are common to all Doppler-based techniques [1]. Two-dimensional speckle tracking echocardiography (STE) is a non-invasive and accurate method for the assessment of left atrial longitudinal myocardial deformation and overcomes the shortcomings of Doppler echocardiography [2–5]. The aim of this study was to elucidate LA function in patients with CP using a combination of speckle-tracking echocardiography (STE) and conventional echocardiography.

## Materials and Methods

### Study participants

Thirty patients (21 men and 9 women) with CP from the First Affiliated Hospital of China Medical University were enrolled in this study between October 2007 and May 2011. The patients with CP included those with heart failure who were diagnosed with CP according to the echocardiographic criteria and evidence of thickened pericardium (minimal pericardial thickness of both lateral sides of the left and right ventricles was more than 3mm by contrast-enhanced CT) or surgically confirmed during pericardiectomy. Respiratory variation in Doppler transmitral early diastolic flow velocity (> 25%) was seen in 25 (83%) patients. There were 14 (47%) patients who underwent computed tomography during preoperative assessment. Five of these patients were found to have pericardial calcifications. All of the patients with CP had sinus rhythm, and none of them had other types of heart disease. The underlying cause of CP was viral pericarditis in 24 (80%) and unknown in 6 (20%) patients. Thirty healthy volunteers (21 men and 9 women), who were matched in age and gender with the study patients were enrolled as controls. Clinical and echocardiographic data were prospectively collected in electronic patient dossier (Neusoft version 4.53; Shenyang, China)

### Ethics

Written informed consent was obtained from all participants, and the study was approved by the China Medical University Ethics Committee.

### Conventional echocardiography studies

While the participants were in the left lateral recumbent position, images were acquired using a Vivid 7 Dimension ultrasound system (GE Healthcare, Waukesha, WI, USA). equipped with a 2-4 MHz phased array probe. All images and measurements were acquired from standard views according to the guidelines of the American Society of Echocardiography, and were digitally stored for offline analysis [6].

The left ventricular (LV) ejection fraction was measured using the biplane modified Simpson’s method and was used as a standard index of global LV systolic function. The ratio between the peak early (E) and late (A) diastolic velocity across the mitral valve was used as a standard index of LV diastolic function. LV longitudinal function was determined by measuring peak systolic velocity (Sa-sep, Sa-lat), peak early diastolic velocity (Ea-sep, Ea-lat), and late diastolic velocity (Aa-sep, Aa-lat) at the level of the mitral septal annulus and lateral annulus on the apical four-chamber view [7].

The LA anteroposterior dimension was obtained from the parasternal long-axis view. The following indices were calculated using the biplane modified Simpson’s method as follows: (1) maximum LA volume (Vmax), measured at the point of the mitral valve opening; (2) preatrial contraction LA volume (Vpre-a), measured at the onset of the P-wave on the simultaneously recorded electrocardiogram; and (3) minimum LA volume (Vmin), measured at the point of mitral valve closure. LA reservoir function was assessed from the filling volume, which was calculated as the Vmax -Vmin; the expansion index, calculated as [LAVmax - LAVmin]/LAVmin) ×100. The LA conduct function was assessed from the the passive atrial stroke volume, which was calculated as LAVmax – LAVpre-a; the passive emptying index, calculated as ([LAVmax – LAVpre-a]/LAVmax) ×100; and the LA conduit volume, calculated as the LV stroke volume – LA filling volume. The LA pump function was assessed from the active atrial stroke volume, which was calculated as LAVpre-a - LAVmin; and the active emptying index, calculated as [LAVpre-a - LAVmin]/LAVpre-a) ×100 [8–10].

### Speckle tracking echocardiography (STE)

Dynamic 2-D ultrasound images of 3 cardiac cycles from apical 4-chamber views were acquired using conventional ultrasound, with a frame rate of 57-72 fps. To measure strain and strain rate, the images were analyzed using customized software within the EchoPAC work station (GE Healthcare). The endocardial boundary of the left atrium was delineated manually, and then the software automatically drew the epicardial boundary. The widths of the regions of interest were adjusted manually to match the actual endocardial and epicardial boundaries. Similar to STE-derived LV analysis, an automatically generated region of interest was divided into 6 segments. LA longitudinal systolic strain and strain rate were calculated from the mean value of the peak systolic strain and strain rate of all LA segments obtained on the four-chamber view during LV systole. The LA longitudinal early diastolic strain rate was calculated from the mean value of the peak early diastolic strain rate of all LA segments obtained on the four-chamber view during LV early diastole. The LA longitudinal late diastolic strain rate was calculated from the mean value of the peak late diastolic strain rate of all LA segments obtained on the four-chamber view during LV late diastole.

### Calibrated integrated backscatter (IBS)

Calibrated IBS evaluates myocardial ultrasound reflectivity, providing an estimate of myocardial structural alterations and fibrotic content [11–13]. The IBS measurements were obtained from the apical 4-chamber view. A 4×4 region of interest was positioned in the myocardium of the middle segment of the LA lateral wall. The measurements of IBS intensity were performed at end-diastole and expressed in decibels (dB). A less negative value indicated a greater myocardial structural alteration.

### Statistical analysis

Statistical analysis was performed using SPSS 17.0 software, and all the measurements are shown as mean ± SD. Data from the patient and control cohorts were compared using an unpaired *t*-test, and Pearson correlation analysis was performed. *P* < 0.05 was considered statistically significant.

## Results

### Characteristics of the study population and LV echocardiographic measurements

A total of 60 patients were analyzed as 2 groups: patients with CP (n = 30) and healthy volunteers (n = 30). The characteristics of the study participants are shown in Table 1. The healthy cohort was both age- and sex-matched to the patients with CP. There was no difference in the body surface area (BSA) between the 2 study groups.

**Table 1:** Characteristics and parameters of conventional echocardiography of patients with constrictive pericarditis (CP) and healthy volunteers (controls).

**Parameters**	**CP**	**Controls**	**P value**
**Clinical characteristics**			
Age	45 ± 15	40 ± 11	NS
Number of men	21/30	21/30	NS
Body surface area	1.65 ± 0.23	1.72 ± 0.19	NS
Systolic blood pressure (mmHg)	113 ± 15	102 ± 20	NS
Diastolic blood pressure (mmHg)	74 ± 11	67 ± 15	NS
Heart rate (beats/min)	92 ± 20	70 ± 11	< 0.001
**Conventional echocardiographic measurements**			
LV septum (mm)	7.48 ± 0.99	7.37 ± 0.62	NS
LV posterior wall (mm)	7.59 ± 0.99	7.22 ± 0.51	NS
LV end-diastolic diameter (mm)	41.51 ± 3.31	46.82 ± 3.96	< 0.001
LV end-systolic diameter (mm)	27.72 ± 3.90	32.49 ± 3.36	< 0.001
LV end-diastolic volume (ml)	59.22 ± 12.62	91.11 ± 17.61	< 0.001
LV end-systolic volume (ml)	22.59 ± 6.75	33.39 ± 7.48	< 0.001
LV stroke volume (ml)	36.63 ± 8.55	57.71 ± 11.66	< 0.001
LV ejection fraction (%)	62.78 ± 4.51	63.36 ± 4.05	NS
Mitral E velocity (m/s)	0.72 ± 0.18	0.76 ± 0.13	NS
Mitral A velocity (m/s)	0.49 ± 0.16	0.62 ± 0.14	0.003
Mitral E/A ratio	1.58 ± 0.51	1.27 ± 0.27	0.011
Sa-sep (cm/s)	7.81 ± 1.11	8.18 ± 1.68	NS
Ea-sep (cm/s)	13.00 ± 3.67	9.93 ± 2.87	0.001
Aa-sep (cm/s)	10.54 ± 3.37	8.43 ± 1.14	0.005
Sa-lat (cm/s)	8.25 ± 2.24	11.39 ± 2.40	< 0.001
Ea-lat (cm/s)	12.57 ± 3.93	14.96 ± 4.04	0.023
Aa-lat (cm/s)	8.78 ± 3.51	9.18 ± 2.04	NS
E/Ea-sep ratio	5.99 ± 2.35	8.12 ± 2.40	0.003
E/Ea-lat ratio	6.46 ± 3.52	5.64 ± 1.46	NS

Values shown are Mean ± SD. LV, left ventricle; E velocity, the peak early diastolic velocity across the mitral valve; A velocity, the peak late diastolic velocity across the mitral valve; peak systolic velocity; Sa-sep, peak systolic velocity at the level of the mitral septal annulus; Ea-sep, peak early diastolic velocity at the level of the mitral septal annulus; Aa-sep, late diastolic velocity at the level of the mitral septal annulus; Sa-lat, peak systolic velocity at the level of the mitral lateral annulus; Ea-lat, peak early diastolic velocity at the level of the mitral lateral annulus; Aa-lat, late diastolic velocity at the level of the mitral lateral annulus.

There was no statistical difference in the LV ejection fraction (LVEF) of the 2 study groups; the LV end-diastolic diameter, LV end-systolic diameter, LV end-diastolic volume (LVEDV), LV end-systolic volume (LVESV) and stroke volume (SV) were significantly decreased in the patients with CP. The E/A ratio was significantly increased in patients with CP. The E/Ea-sep ratio was significantly decreased in patients with CP, and there was no significant difference in the E/Ea-lat ratios (Table 1).

**Table 2:** Left atrial parameters of patients with constrictive pericarditis (CP) and healthy volunteers (controls).

**Parameters**	**CP**	**Controls**	**P value**
LA anteroposterior diameter (mm)	40.14 ± 6.23	31.28 ± 3.74	< 0.001
LAVmax (ml)	56.23 ± 19.52	39.93 ± 8.32	< 0.001
LA Vmax/BSA (ml/m^2^)	34.04 ± 15.25	23.53 ± 5.16	0.006
LA Vpre-a (ml)	37.12 ± 15.92	22.17 ± 7.68	< 0.001
LA Vpre-a/BSA(ml/m^2^)	22.79 ± 12.55	13.07 ± 4.76	0.002
LA Vmin (ml)	28.23 ± 15.53	13.59 ± 4.87	< 0.001
LA Vmin /BSA (ml/m^2^)	17.48 ± 12.11	7.99 ± 2.89	0.002
Filling volume (ml)	28.00 ± 7.42	26.36 ± 5.29	NS
Filling volume/BSA(ml/m^2^)	16.56 ± 4.91	15.54 ± 3.43	NS
Expansion index	116.43 ± 45.21	211.23 ± 71.22	< 0.001
Passive atrial stroke volume (ml)	19.12 ± 7.14	17.79 ± 4.68	NS
Passive atrial stroke volume/BSA(ml/m^2^)	11.25 ± 4.58	10.46 ± 2.85	NS
Passive emptying index	34.82 ± 9.59	45.25 ± 10.72	< 0.001
Active atrial stroke volume (ml)	8.88 ± 3.24	8.57 ± 3.99	NS
Active atrial stroke volume/BSA(ml/m^2^)	5.32 ± 2.22	5.07 ± 2.49	NS
Active emptying index	26.12 ± 10.22	37.85 ± 10.47	< 0.001
LA conduit volume (ml)	9.04 ± 10.56	31.36 ± 12.59	< 0.001
Calibrated integrated backscatter (dB)	-27.15± 3.49	-29.73± 3.65	0.035

Values shown are Mean ± SD. LA, left atrium; LAVmax, maximum left atrial volume; LAVpre-a(Vmax), preatrial contraction left atrial volume; LAVmin, minimum left atrial volume; BSA, body surface area.

### LA systolic and diastolic function of patients with CP

**Figure 1:**
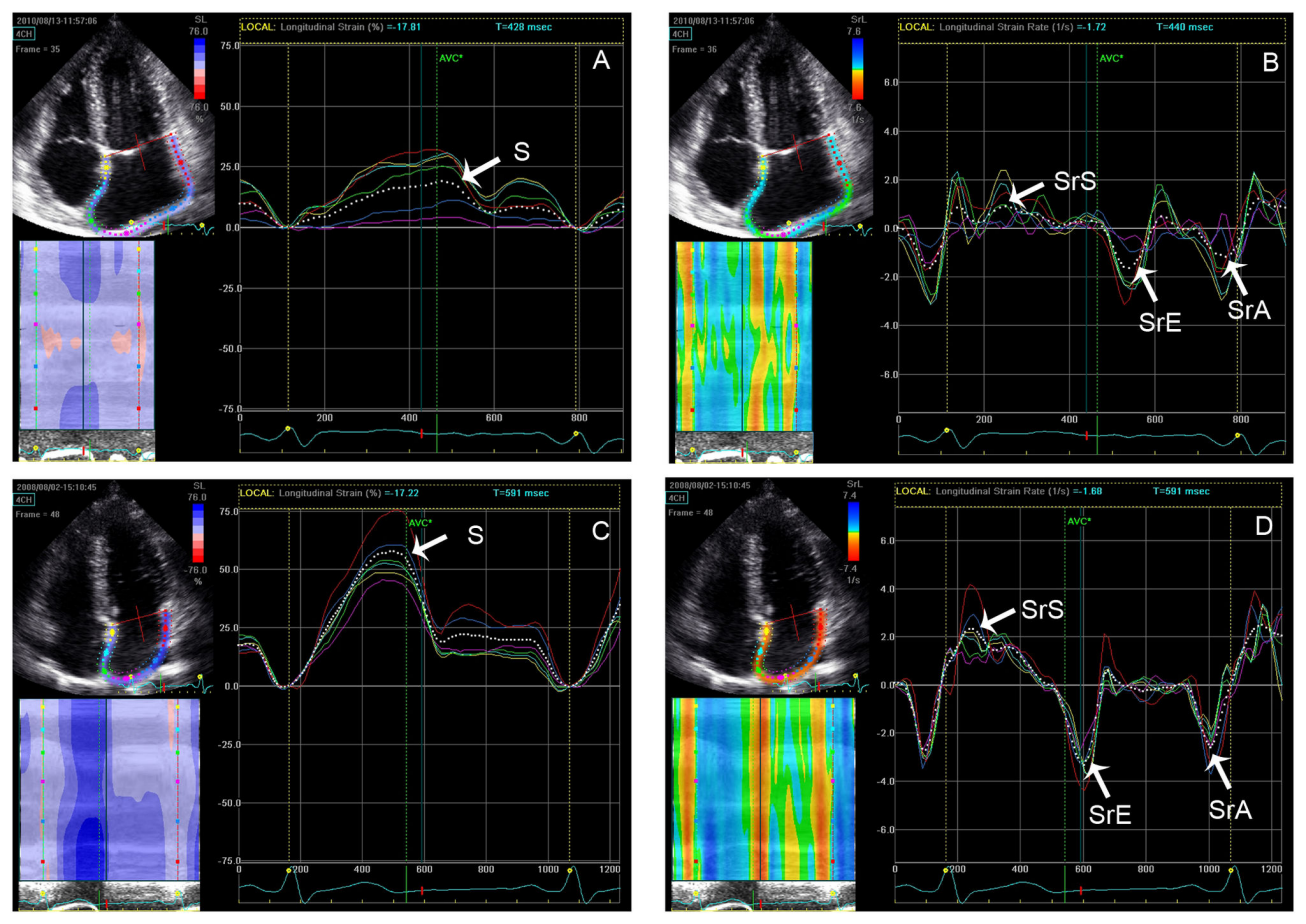
LA strain and strain rate in patients with constrictive pericarditis (A, B) and healthy controls (C, D).

**Figure 2:**
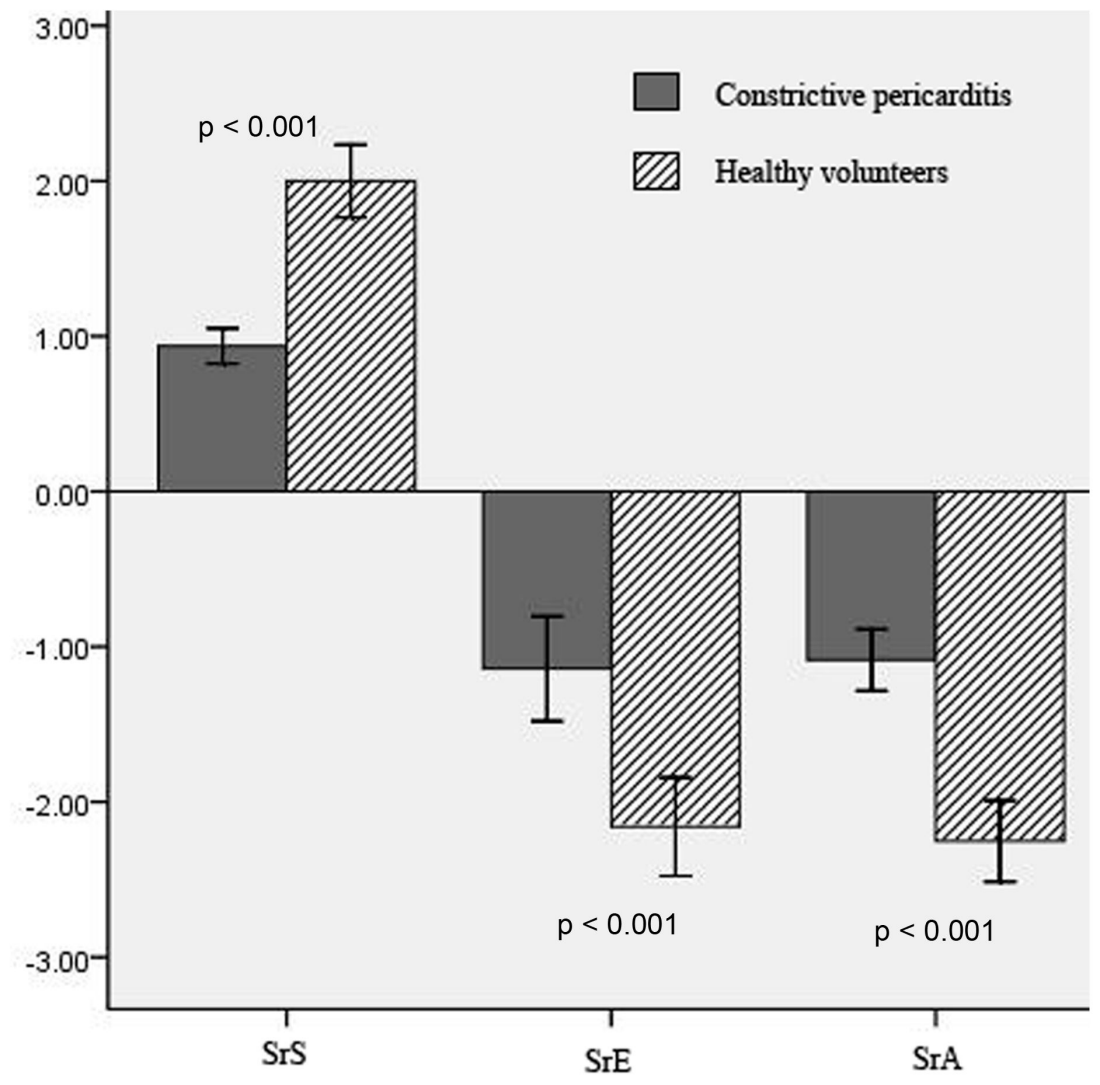
Differences in atrial systolic and diastolic strain rate in patients with constrictive pericarditis and healthy controls.

**Figure 3:**
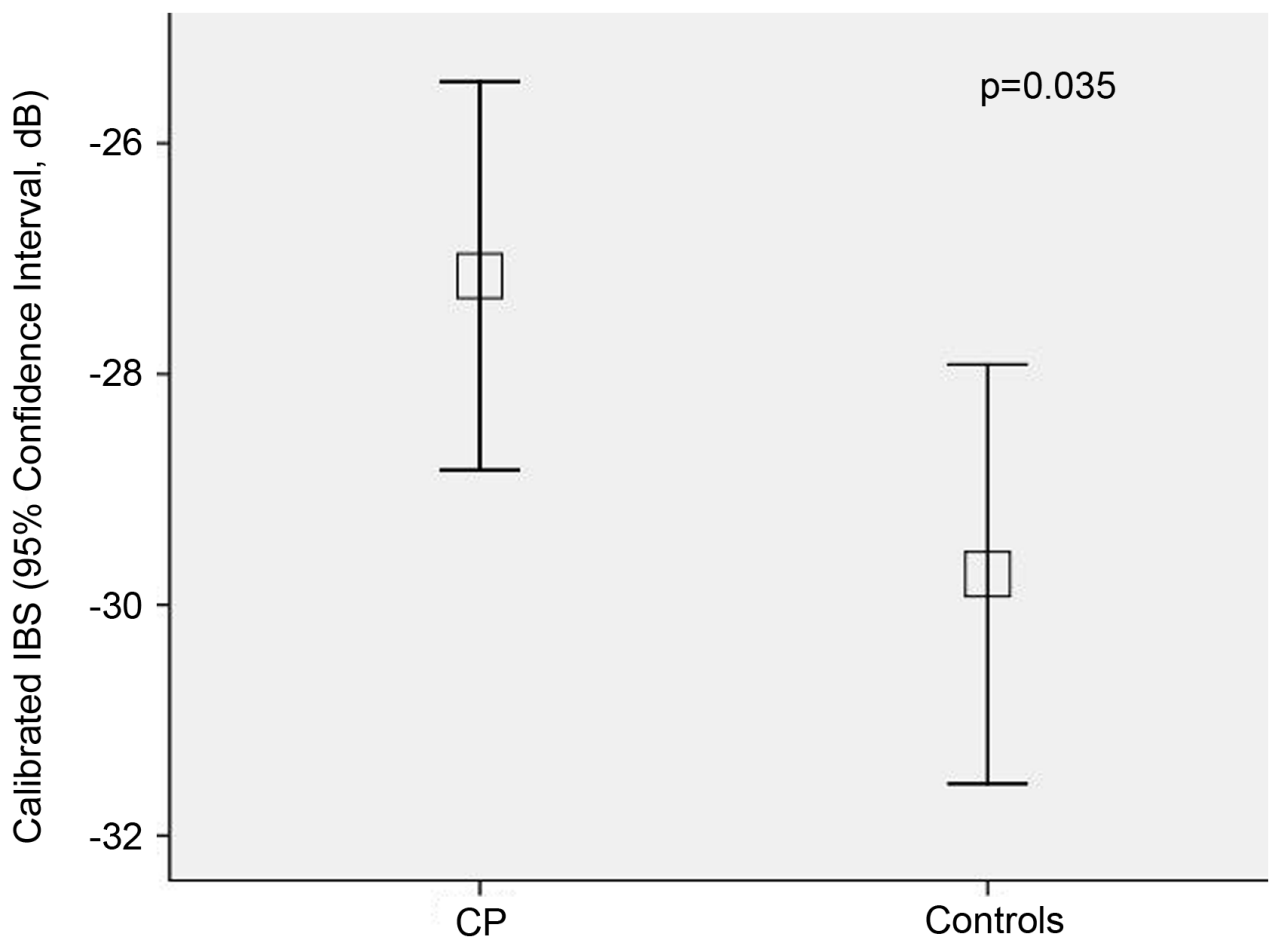
Calibrated integrated backscatter (IBS) of the LA lateral wall in patients with constrictive pericarditis (CP) and healthy controls.

Values for LA diameter and function are shown in Tables 2 and 3. The LA anteroposterior diameter, the Vmax, Vpre-a, and Vmin were significantly increased in patients with CP, although the filling volume in patients with CP was not significantly decreased. However, the LA reservoir function, as shown by the expansion and diastolic emptying indices, was reduced in patients with CP. There was no significant difference in the passive atrial stroke volume between the 2 study groups; however, the passive emptying index and LA conduit volume were significantly decreased in patients with CP, which indicates that the conduit function in patients with CP was reduced. Although the LA active atrial stroke volume was similar between the 2 study groups, the active emptying index was significantly decreased in patients with CP. STE demonstrated that the global S, SrS, SrE, and SrA were significantly decreased in patients with CP (Figures 1 and 2). In addition, structural alteration in the LA lateral wall region evaluated by calibrated IBS was higher in patients with CP (-27.15± 3.49) as compared to the healthy cohort (-29.73± 3.65, *P* = 0.035. Figure 3).

**Table 3:** Parameters of speckle tracking echocardiography of patients with constrictive pericarditis (CP) and healthy volunteers (controls).

**Parameters**	**CP**	**Controls**	**P value**
S	16.53 ± 5.46	44.96 ± 16.18	< 0.001
SrS	0.90 ± 0.26	2.09 ± 0.66	< 0.001
SrE	-1.30 ± 0.44	-2.27 ± 0.85	< 0.001
SrA	-1.06 ± 0.53	-2.30 ± 0.78	< 0.001

Values shown are Mean ± SD. S, the left atrial global systolic strain; SrS, the left atrial global systolic strain rate; SrE, the left atrial global early diastolic strain rate; SrA, the left atrial global late diastolic strain rate.

### Correlation analysis

In patients with CP, the E/Ea-lat ratio was positively correlated with the global SrE (*r* = 0.478, *P*=0.013) and negatively correlated with the global SrS (*r* = 0. -516, *P*=0.007). Moreover, the structural alteration in the LA lateral wall region evaluated by calibrated IBS was positively correlated with the global SrE (*r* = 0.405, *P*=0.024). The E/Ea-sep was not correlated with the STE values. The correlation between SrA and Ea-lat was significant (*r* = -0.399 *P* = 0.032), whereas the SrA not significantly correlated with the Ea-sep. The mitral E/A was not correlated with the STE values, and there was also no significant correlation between the LVEF and STE values.

## Discussion

There are 4 components of normal LA function: (1) as a reservoir during LV systole and isovolumic relaxation; (2) a conduit for blood transiting from the pulmonary veins to the left ventricle during early diastole; (3) an active contractile chamber that establishes final LV end-diastolic volume in late diastole; and (4) a suction source that refills itself in early systole [14,15]. In normal individuals, the relative contributions of the LA reservoir, conduit, and contractile functions to the filling of the left ventricle are approximately 40%, 35%, and 25%, respectively [16]. LA function plays an important role in the maintenance of cardiac output, and impairment of LA function can lead to circulatory failure, mitral regurgitation, atrial fibrillation, and stroke [17,18].

Some investigators have demonstrated that LA function is an important predictor of cardiovascular events, and is independently associated with the risks for stroke and death [19,20]. The LA longitudinal strain and strain rate, which inversely related to LA wall fibrosis has been reported to be a feasible and reproducible method to assess LA myocardial function [2,16,21,22]. Recent researches have shown that LA strain is an independent predictor of the occurrence of LA remodelling and concomitant deterioration of LA function during followup [23–25]. In addition, LA strain has been related to LA structural remodelling and fibrosis of the atrial wall as assessed with magnetic resonance imaging [22]. Diastolic dysfunction from disorders such as hypertension, type 2 diabetes, obesity, and coronary artery disease is characterized by the subendocardial dysfunction of both the left atrium and the left ventricle [4]. However, whether pericardial restriction and adhesion can lead to both systolic and diastolic dysfunction of the left atrium, and the characteristics of LA function in patients with CP remain unclear. To the best of our knowledge, this is the first study to describe LA function in patients with CP using speckle tracking and conventional echocardiography.

Previous studies have suggested that a Frank-Starling mechanism also exists in the human left atrium [26–28]. In diastolic dysfunction resulting from hypertension, coronary artery disease, diabetes mellitus, and advanced age, LA pump function was found to improve in response to the increased LAVpre-a and an enhanced inotropic state of the LA myocardium [29,30]. In patients with CP, as the pericardial restriction of the LV, the LA pressure in raises to maintain adequate LV filling pressure, and the increased atrial wall tension leads to chamber dilatation and stretch of the atrial myocardial. In the study, although the active LA stroke volume was not decreased in patients with CP, the active emptying index and the SrA, as measured by LA strain, were decreased, indicating that the contractility function of the LA myocardium was not enhanced, and that the deformation of the LA myocardium was reduced.

During left ventricular contraction, LA reservoir function is determined by LA myocardial contraction and relaxation and displacement of the mitral annulus [14]. In this study, the LA expansion index, S, and SrS were decreased in patients with CP in comparison with the healthy volunteers, indicate that the LA reservoir function was decreased in patients with CP, in other words, the LA passive enlargement was impaired during atrial filling phase.

LA conduit function is mainly determined from the rate of LV relaxation [31]. In this phase, blood flows from the pulmonary veins into the left atrium, then from the left atrium into the left ventricle. Hiroyama et al have demonstrated that in patients with CP, the active shortening and passive enlargement of the LA are decreased, and the atrial tend to be a conduit [32]. However, in this study, we found in patients with CP, the conduit volume and the SrE were also markedly reduced, in addition to the decreased reservoir and pump function, this change may attribute to the pericardial restriction of the LV and the organic change in the atrial muscle.

Recently, E/Ea has been proposed as a tool for assessing LV filling pressures. Several investigators have shown that in patients with either impaired or pseudonormal relaxation or with sinus tachycardia, E/Ea is positive correlated with pulmonary artery or pulmonary capillary wedge pressure (PCWP) [33,34]. However, in patients with CP, the E/Ea-sep is inversely correlated with the PCWP, because the Ea is usually well preserved or even accentuated despite increased filling pressure, and Ea from the lateral annulus may be affected by calcifications or adhesions of the pericardium [35]. In the study, the LA values did not correlated to the LV diatolic and systolic function evaluated by mitral E/Ea-sep and LVEF, indicate that elevated filling pressure caused by the pericardial restriction of the LV is not the only reason of atrial dysfunction in patients with CP. Moreover, in patients with CP, we found the E/Ea-lat ratio was positively correlated with the global SrE (*r* = 0.478, *P*=0.013) and negatively correlated with the global SrS (*r* =-0.516, *P*=0.007). In addition, the correlation between SrA and Ea-lat was significant (*r* = -0.399 *P* =0.032) was found in patients with CP, suggesting that LA myocardial function is potentially affected by the pericardium, because the Ea-lat is potentially affected by pericardial calcifications or adhesions [35].

Several studies have demonstrated that IBS correlated with the collagen content of the myocardium [36,37]. Therefore, this technique is thought to provide an noninvasive estimation of myocardial fibrosis in humans [38]. In the study, the structural alteration in the LA lateral wall region evaluated by calibrated IBS was predominant in patients with CP as compared to the healthy cohort, and positively correlated with the global SrE (*r* = 0.405, *P*=0.024) in patients with CP, suggested the myocardial fibrotic changes may play an important role in the reduction of LA function.

A major limitation of this study was the lack of invasive hemodynamic data, primarily because invasive data was not clinically indicated [39]. Several studies have shown that E/Ea has high sensitivity, specificity, and accuracy to determine the LV filling pressures [33–35,40]. Therefore, the guidelines of the American Society of Echocardiography for determining LV diastolic function recommend the noninvasive estimation (E/Ea) of LV filling pressures [41], however, the justification is not valid as a measurement of filling pressure in patients with CP. Moreover, there is no followup of these patients. Furthermore, the LA strain measurements in two-chamber view was not investigated because of its low reproducibility and because the corresponding data post-processing is both complex and time consuming [23], that could limit the results of the study. Additionally, in this study, LA function in patients with CP and healthy volunteers were evaluated in this study, the major differential diagnostic disease of CP was restrictive cardiomyopathy (RCM), which was not enrolled in the study. We have studied some cases of RCM, and did found a difference between RCM and CP, further studies with more RCM patients are planned to assess.

## Conclusions

In this study, the LA systolic and diastolic function of patients with CP were found to be reduced, the pericardial restriction and LA myocardial impairment resulting from fibrotic changes and inflammation of the pericardium may play an important role in the reduction of LA function.
